# Nanoparticle-based ‘turn-on’ scattering and post-sample fluorescence for ultrasensitive detection of water pollution in wider window

**DOI:** 10.1371/journal.pone.0227584

**Published:** 2020-01-09

**Authors:** Soumendra Singh, Animesh Halder, Oindrila Sinha, Probir Kumar Sarkar, Priya Singh, Amrita Banerjee, Saleh A. Ahmed, Ahmed Alharbi, Rami J. Obaid, Sanjay K. Ghosh, Amitabha Mitra, Samir Kumar Pal

**Affiliations:** 1 Department of Chemical, Biological and Macromolecular Sciences, S.N Bose National Centre for Basic Sciences, Kolkata, West Bengal, India; 2 Centre for Astroparticle Physics and Space Science, Bose Institute, West Bengal, Kolkata, India; 3 Technical Research Centre, S. N. Bose National Centre for Basic Sciences, Kolkata, West Bengal, India; 4 Department of Applied Optics & Photonics, University of Calcutta, Kolkata, West Bengal, India; 5 Department of Life Sciences, Presidency University, Kolkata, West Bengal, India; 6 Department of Physics, Ananda Mohan College, Kolkata, West Bengal, India; 7 Department of Chemistry, Faculty of Applied Sciences, Umm Al-Qura University, Makkah, Saudi Arabia; Institute of Materials Science, GERMANY

## Abstract

Ultrasensitive detection of heavy metal ions in available water around us is a great challenge for scientists since long time. We developed an optical technique that combines Rayleigh scattering of UV light (365 nm) and post-sample fluorescence detection from colloidal silver (Ag) nanoparticles (NPs) having a surface plasmon resonance (SPR) band at 420 nm. The efficacy of the technique is tested by the detection of several model toxic ions, including mercury, lead, and methylmercury in aqueous media. The light scattering from the Hg-included/inflated Ag NPs at 395 nm was observed to saturate the light sensor even with ppm-order concentrations of Hg ions in the water sample. However, the pollutant is not detected at lower concentrations at this wavelength. Instead, the fluorescence of a high-pass filter (cut-off at 400 nm) at 520 nm is applied to detect pollutant concentrations of up to several hundreds of ppm in the water sample. We also detected lead and methylmercury as model pollutants in aqueous media and validated the efficacy of our strategy. Finally, we report the development of a working prototype based on the strategy developed for efficient detection of pollutants in drinking/agricultural water.

## Introduction

Heavy metal ion pollution is a severe problem threatening the environment, human health, and ecosystem balance[1]. Some of the most hazardous and ubiquitous pollutants that threaten the integrity of the ecosystem and have a deleterious effect on the health of humans and future generations are Mercury(II) (Hg^2+^) and its organic form (i.e. methylmercury)[2] and Lead(II) (Pb^2+^)[1,3]. Hg^2+^ can easily permeate through biological membranes and the blood-brain barrier and can damage DNA, impair cell division, and cause neurological damage[1,4,5]. The more potent form of Hg^2+^ is its organic form, i.e. methylmercury[6]. Thousands of individuals were affected by the Minamata disaster in Japan, one of the first large-scale accidents of methylmercury poisoning and several other such accidents in other parts of the world affected many others[7]. Methylmercury has higher potency due to its high lipid solubility, which aids it in bio-accumulation and bio-amplification using the food chain of the ecosystem[8]. Mercury and methylmercury are dangerous neurotoxins, which are particularly hazardous for infants and pregnant women [9,10].

Although the Minamata accident raised global concern regarding the detrimental effects of mercury, the ill-effects of heavy metals, including mercury, were known to mankind previously. Mercury is categorised as a neuro-toxicant, powerful and dangerous to foetuses and children because it has the ability to pass through the placenta[11]. Even decades after the industrial dumping, survivors of mercury-pollution accidents still suffer from acute intoxication and its consequences[7]. The high toxicity of mercury and other heavy metal ions, even in trace amounts, calls for a very specific and highly sensitive determination. Sensitive detection of Mercury will lead to more effective toxicological, environmental, and biological monitoring.

The established techniques for monitoring and determining the Hg^2+^ concentration in environmental samples include atomic absorption/emission spectroscopy, X-ray absorption spectroscopy, inductively coupled plasma mass spectrometry (ICPMS), and surface-enhanced Raman scattering (SERS)[12–17]. However, these techniques require sophisticated and expensive instrumentation and are highly time-consuming. Recently, a variety of ‘turn-on’ spectroscopic methods have been reported and used; these include fluorescence and colourimetry, which are advantageous for analysing complex biological samples and monitoring dynamic biological processes in living cells[18–23]. Great potential in the sensitive detection of Mercury is displayed by various fluorescent probes, such as semiconductor quantum dots and organic molecules [24–26]. However, the practical application of these fluorescent probes is hindered by their complex synthesis routes and the expensive and toxic reagents that are employed. The turbidity of the sample also interferes with the testing and determination of the concentration of heavy metal ions in solution. Recently, few studies have been carried out using the wavelength and size-dependent ‘Faraday-Tyndall’ effect in gold nanoparticles (NPs) and the associated surface plasmon resonance (SPR) phenomena [27]. In a recent study, laser-induced microbubbles (LIMBs) in gold colloid were reviewed, and their various properties including scattering and SPR were investigated in detail[28]. However, instances of heavy-ion detection in water are sparse in literature and few sensors have been developed based on the ‘Faraday-Tyndall’ effect. Sensitive detection of other metal ions like Aluminium has also been explored and its efficacy in presence of other metal ions with water-soluble onion-like carbon nanoparticles (wsCNOs) was reported [29]. Various nano carbon based sensors having substantial potential in detection of toxins, microbes, heavy metal ions, inorganic and organic pollutants in water were reviewd recently [30]. α-FeOOH nanoparticles have also been used to extract Arsenic from contaminated water [31].

Contemporary sensors of environmental pollutants can be classified into two categories: higher sensitivity with a limited detection window and lower sensitivity with a wider detection window. Measurement strategies of real-world samples are often associated with complicated sample preparation in order to comply with the requirements of conventional sensors [32], which sometimes becomes cumbersome in field-based measurement setting. It is preferable to have multiple parameters to monitor pollutants with higher sensitivity in wider measurement windows [33]. These can originate from different physical phenomena due to the interaction of the target pollutant with the sensor. Here, we developed a strategy of monitoring environmental water pollutants based on the enhanced Rayleigh scattering upon interaction of the target pollutant with the NPs of the sensor and post sample fluorescence from a high-pass (HP) optical filter. Citrate-functionalized silver (Ag) NPs with an SPR band at 420 nm are used as a model sensor and the detection of a variety of water pollutants, including mercury ions, lead, and methylmercury, is tested to justify the efficacy of the proposed strategy. Using our strategy, we also designed a cost-effective, user-friendly, and highly efficient device based on an optical technique, combining the principles of ‘turn-on’ Rayleigh scattering and post sample fluorescence from an optical filter in a wider detection window. Our device is shown to be useful for detecting heavy metal ions (mercury, methylmercury, and lead) with very high sensitivity in a wider detection window and with high selectivity as reported in one of the previous publication from or group [34]. The merits and demerits of all existing techniques for detection of heavy metal ions in available water samples in comparison to our developed strategy has been presented in a tabular form in Table 1.

**Table 1:** Comparative representation of available techniques for heavy metal ion detection vis-à-vis our developed strategy.

Methods	High Sensitivity	Low cost	Portability	Reproducibility	Stability	High dynamic range	Ease of sample preparation
**1.Electro-chemical Sensors** **(e.g. Potentiometric, Amperometric, Conductometric, Ion selective FET etc.)**	✗	✗	✓	✗	✗	✗	✓
**2. Optical** **(e.g. Absorbance Sensors, SPR, Fluorescence Sensors, Luminescence Sensors etc.)**	✓	✗	✗	✓	✓	✓	✓
**3. Piezoelectric Sensors**	✗	✓	✓	✗	✗	✗	✓
**4.Thermal Sensors**	✗	✓	✓	✗	✗	✗	✗
**5. Enzyme/Antibiotic Sensors**	✓	✓	✗	✓	✓	✗	✗
**6. MS** **ICPMS, OES**	✓	✗	✗	✓	✓	✓	✗
**7. Turn ON Rayleigh scattering and post sample fluorescence method**	✓	✓	✓	✓	✓	✓	✓

## Materials and methods

All chemicals used are of analytical grade. No further purification of the chemicals was performed. Silver nitrate (AgNO_3_, 99.99%), sodium borohydride (NaBH_4_), sodium citrate (Na_3_C_6_H_5_O_7_), and sodium hydroxide (NaOH) were purchased from Sigma-Aldrich. The samples were in the form of nitrate or chloride salts, which were used as received from Merck and Aldrich without further purification. A known amount of chloride or nitrate salts were used in the aqueous medium to prepare the stock solution (50 mM) of the metal ions. Millipore water (from Merck) was used as the aqueous solvent.

### Synthesis of Ag NPs

A process including the reduction of AgNO_3_ with NaBH_4_ resulted in the synthesis of nitrate-capped Ag NPs at pH = 6.5. Initially, we prepared a quick mixture containing stock solutions of 1 mM sodium citrate and 5 mM of both NaBH_4_ and AgNO_3_ in aqueous solution. A stirrer was used for the smooth amalgamation of 16 mL of 1 mM solution in water and 1 mL aqueous AgNO_3_ solution, maintained at a temperature of 0°C with an ice bath. Drops of instantly produced aqueous NaBH_4_ (150 mL of 5 mM) solution were added for 5 min. A distinct shift of the solution from colourless to intense yellow was observed. This solution was stirred for 2.5 h. An absorption peak at 420 nm (SPR spectroscopic signature) from the X-ray absorption spectra confirmed the successful synthesis of the Ag NPs with the above-mentioned process. [34,35].

## Development of the detection setup

**Fig 1:**
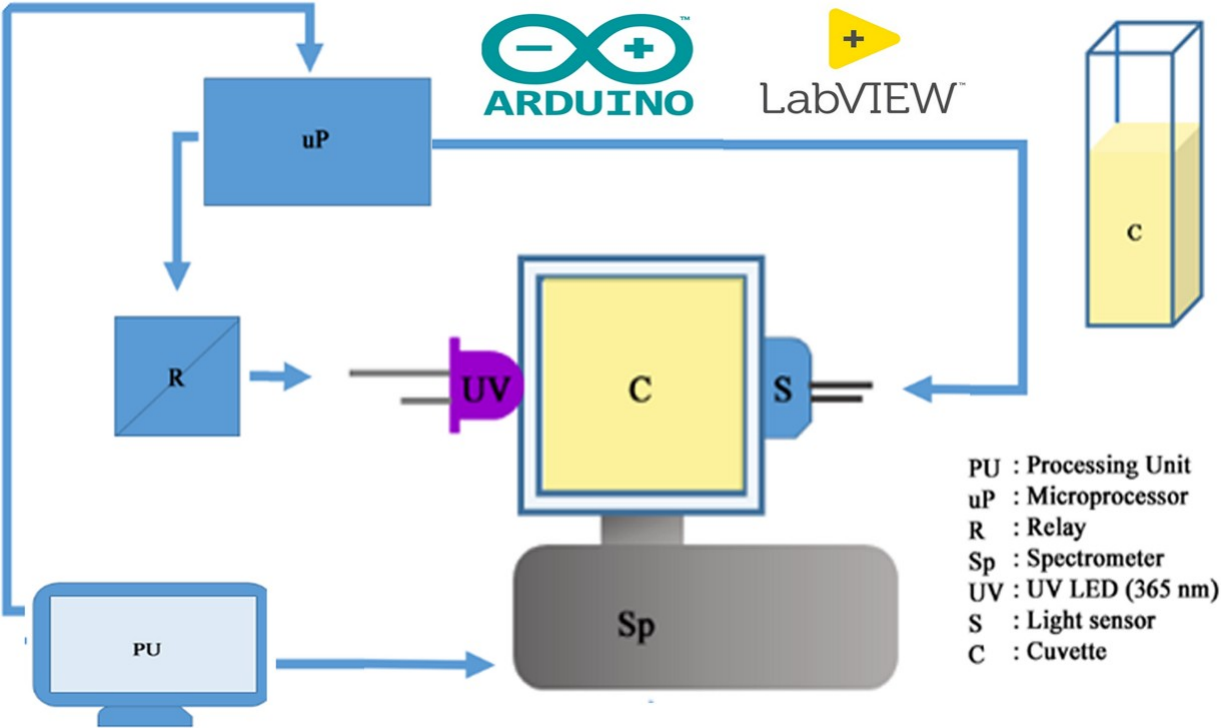
Electro-optical set-up of the proposed device, which works on the principle of ‘turn-on Rayleigh Scattering’.

**Fig 2:**
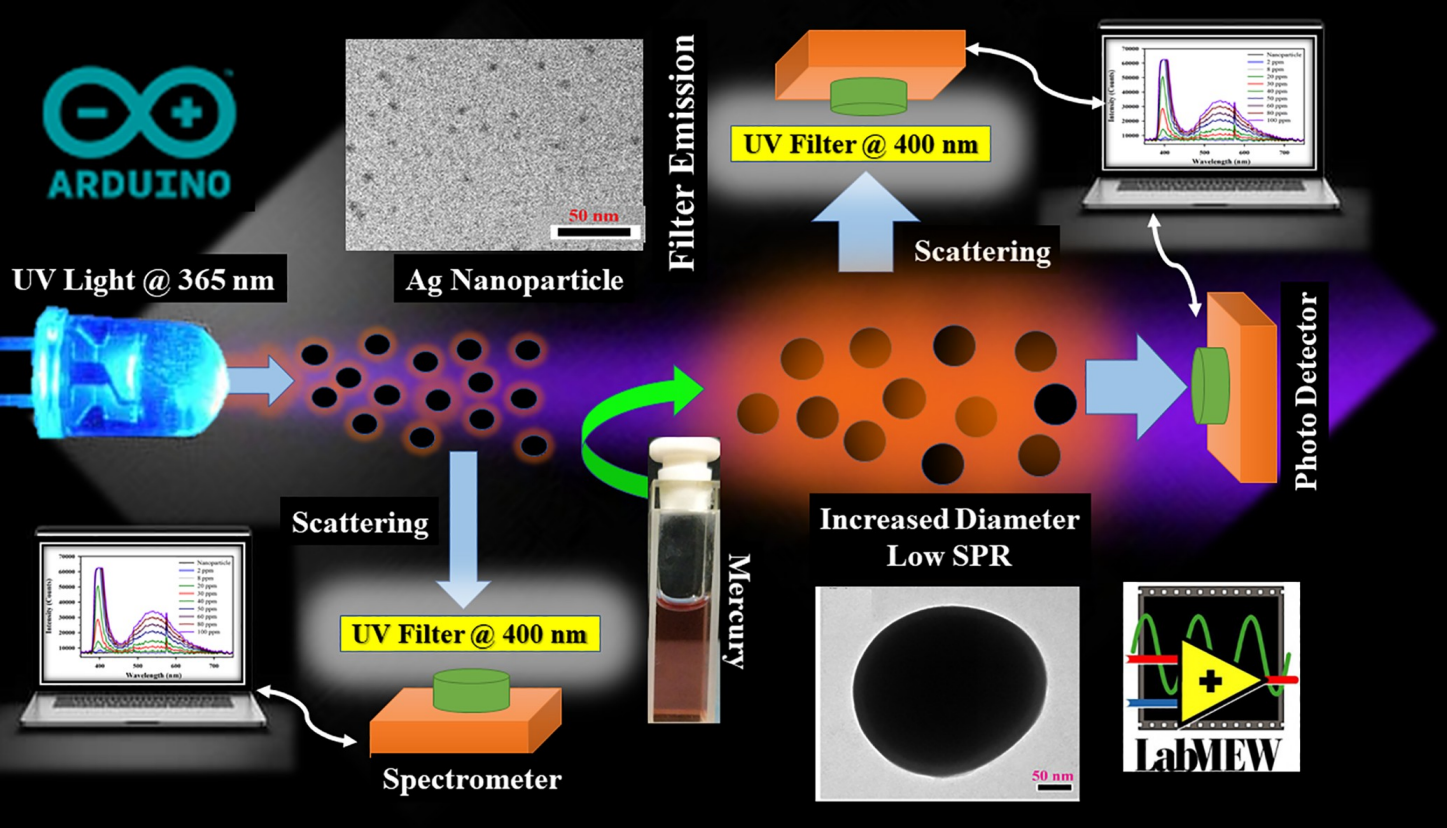
Schematic of ‘turn-on’ scattering sensor for highly sensitive detection of pollutants (Mercury in this study). (see text).

The detection system consisted of three basic components: a Light Emitting Diode (LED) source, a customised cuvette holder, and the detector. We used a 3 W Ultra Violet (UV) LED with 365 nm wavelength (Ocean Optics, Florida, USA) and a Charged Couple Device (CCD)-based detector (Black-Comet, C-SR-200, StellarNet Inc., USA). The cuvette holder was designed in such a way that the detector and the LED remain orthogonal to each other. An optical filter (Ocean Optics, Florida, USA) with passband > 400 nm was placed in front of the detector. In this setup, the light passing through the customised cuvette holder excites the sample and, after being scattered, is collected by the detector. A microcontroller (Arduino Uno) was used to control the LED through a solid-state relay. The overall algorithm, including data acquisition, data processing, data analysis, and decision making, was controlled by a self-developed LabVIEW-based software. Fig 1 presents a schematic of the self-developed low-cost electro-optical set-up based on ‘turn-on’ Rayleigh scattering and post sample fluorescence. The UV light excites the sample, and the emission is collected at an angle of 90° relative to the incident light beam by the spectrometer; the absorption of the sample is collected by an array detector at an angle of 0° relative to the excited light beam. After passing through the sample, the 465 nm scattered light reaches the filter, which emits a broadband fluorescence light spectrum. An HP filter (λ_pass_ > 400 nm) is used to split the excitation light beam and provide broad-spectrum fluorescence. The HP filter acts as a secondary detector as the scattered light saturates the receiver quickly due to increased diameter of the Ag NP in the mercury solution. Fig 2 presents a schematic of the ‘turn-on’ scattering sensor for the high-sensitive detection of pollutants (Mercury here). UV light (365 nm) from an LED hits the Ag NPs with an SPR absorption at 420 nm. In the absence of mercury, the NPs exhibit significant absorption of the excitation light and scatter the light very weakly to the HP filter in front of the spectrometer. The light scattered by the HP filter and its fluorescence are shown to peak at 395 and 520 nm, respectively. In the presence of mercury, the NPs agglomerate and the overall size of the particles increases, decreasing the SPR absorption and enhancing the light scattering to the HP filter, which eventually increases the fluorescence intensity of the filter.

### Software design

**Fig 3:**
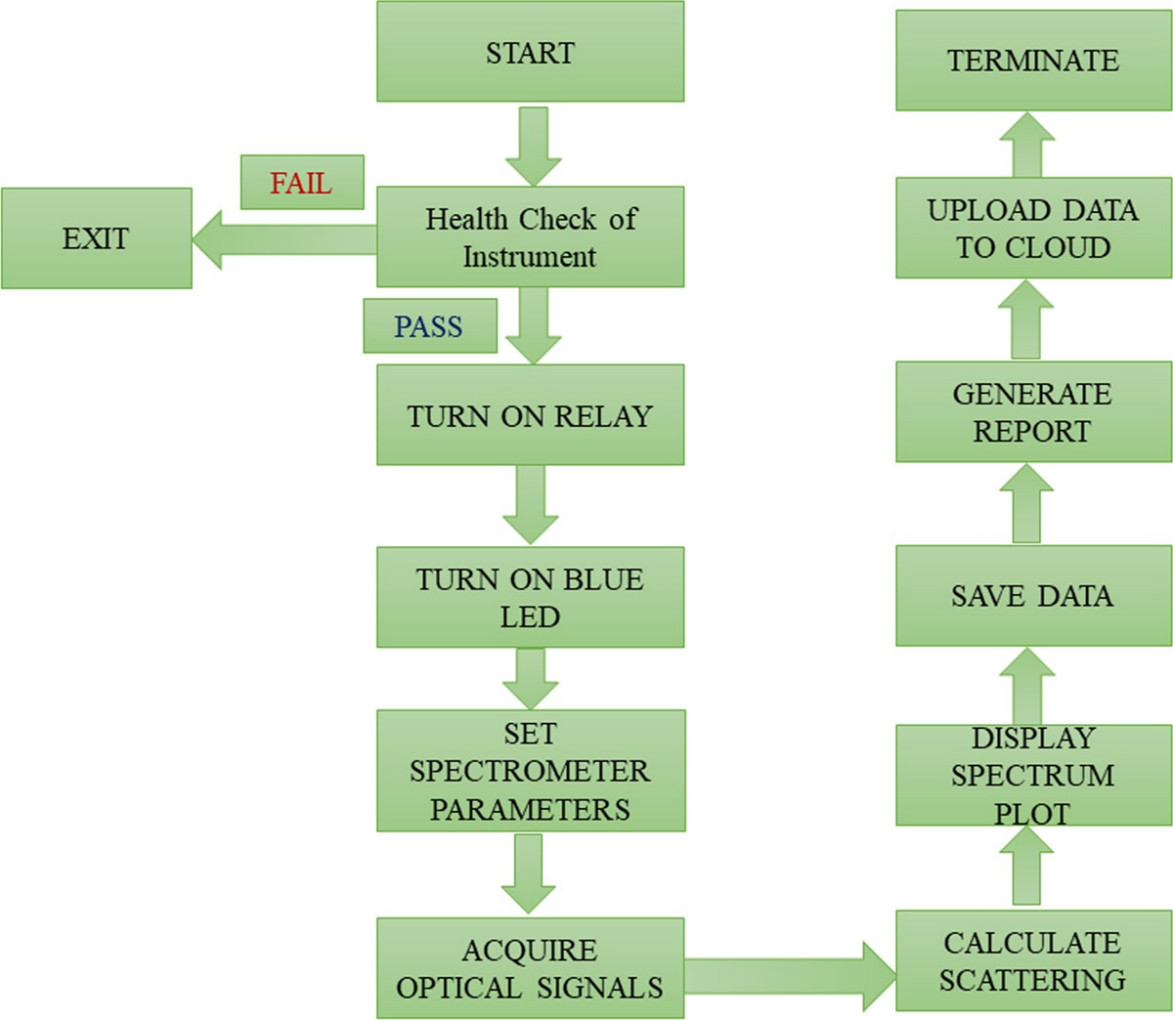
Workflow/algorithm of the self-developed software to control the device with a proper graphical user interface.

We developed a user-friendly LabVIEW-based Graphical User Interface (GUI) for data acquisition and real-time analysis of the results. The software acquires data sequentially by turning on the LED light, setting the spectrograph parameters, including the wavelength range, integration time, and acquisition interval, and analysing the data. Initially, the software checks the health of various components of the instrument. Subsequently, it turns on the blue LED and initialises the data acquisition; it displays a graph on the computer screen and stores all desired parameters as American Standard Code for Information Interchange (ASCII) files at the desired location. Fig 3 illustrates the workflow/algorithm of the self-developed software to control the device with proper graphical user interface.

### Optical studies

A Shimadzu 2600 absorption spectrometer was used to conduct the optical density (OD) spectrometric studies of the films. Water baseline was performed, and data were used as reference for all OD observations. The particle size and morphology before and after interaction with the heavy metal ions were determined using (HRTEM) high-resolution transmission electron microscopy (FEI Technai S-Twin) with an acceleration voltage of 200 kV. Copper grids coated with carbon were drop-casted with dilute samples to prepare the thin films necessary for HRTEM.

## Results and discussion

### Interaction of Ag NP with heavy metal ions

**Fig 4:**
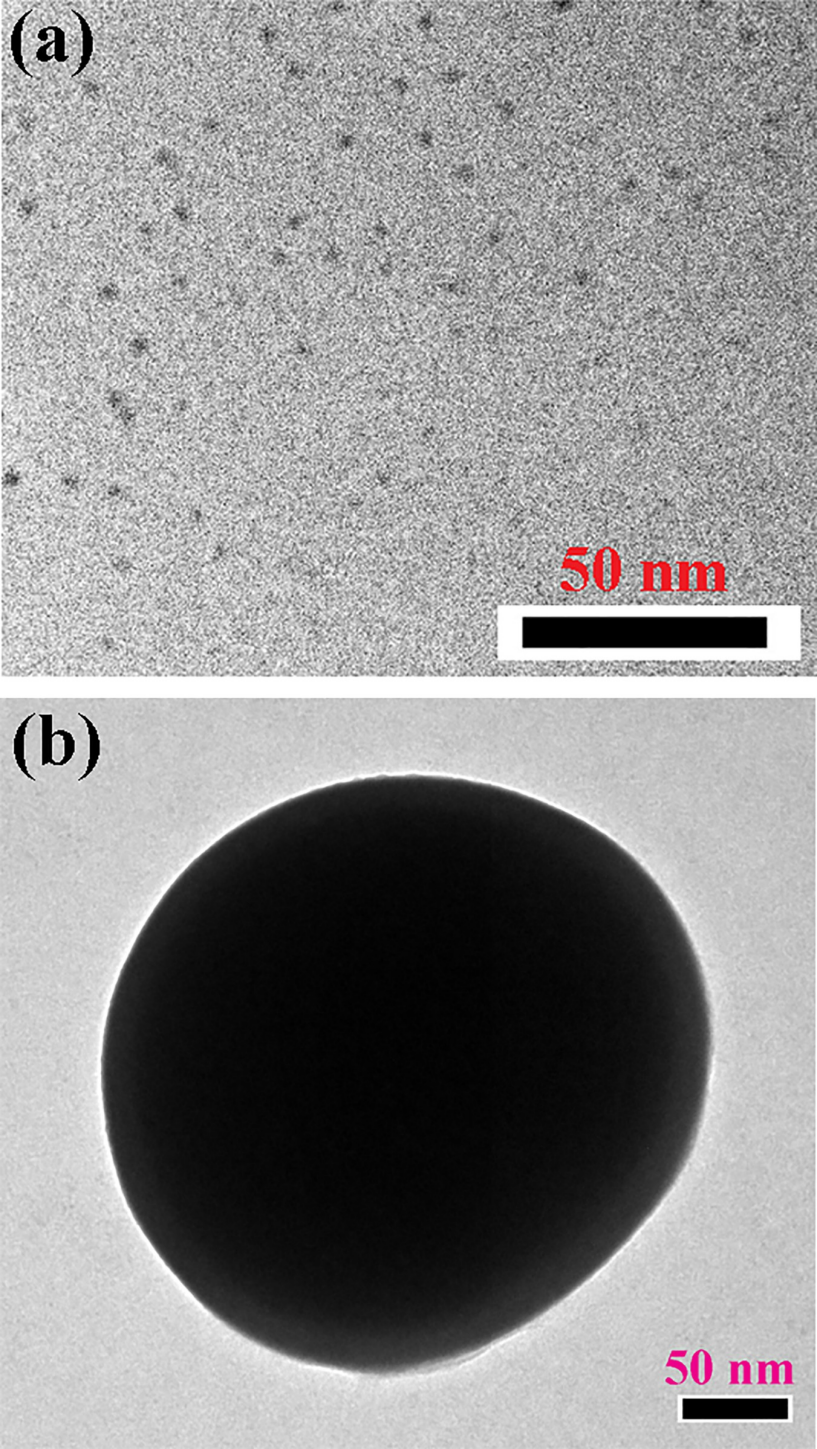
HRTEM images of Ag NPs (a) before and (b) after interaction with mercury ions in aqueous environments. Diameter change is due to Hg^2+^-induced agglomeration.

**Fig 5:**
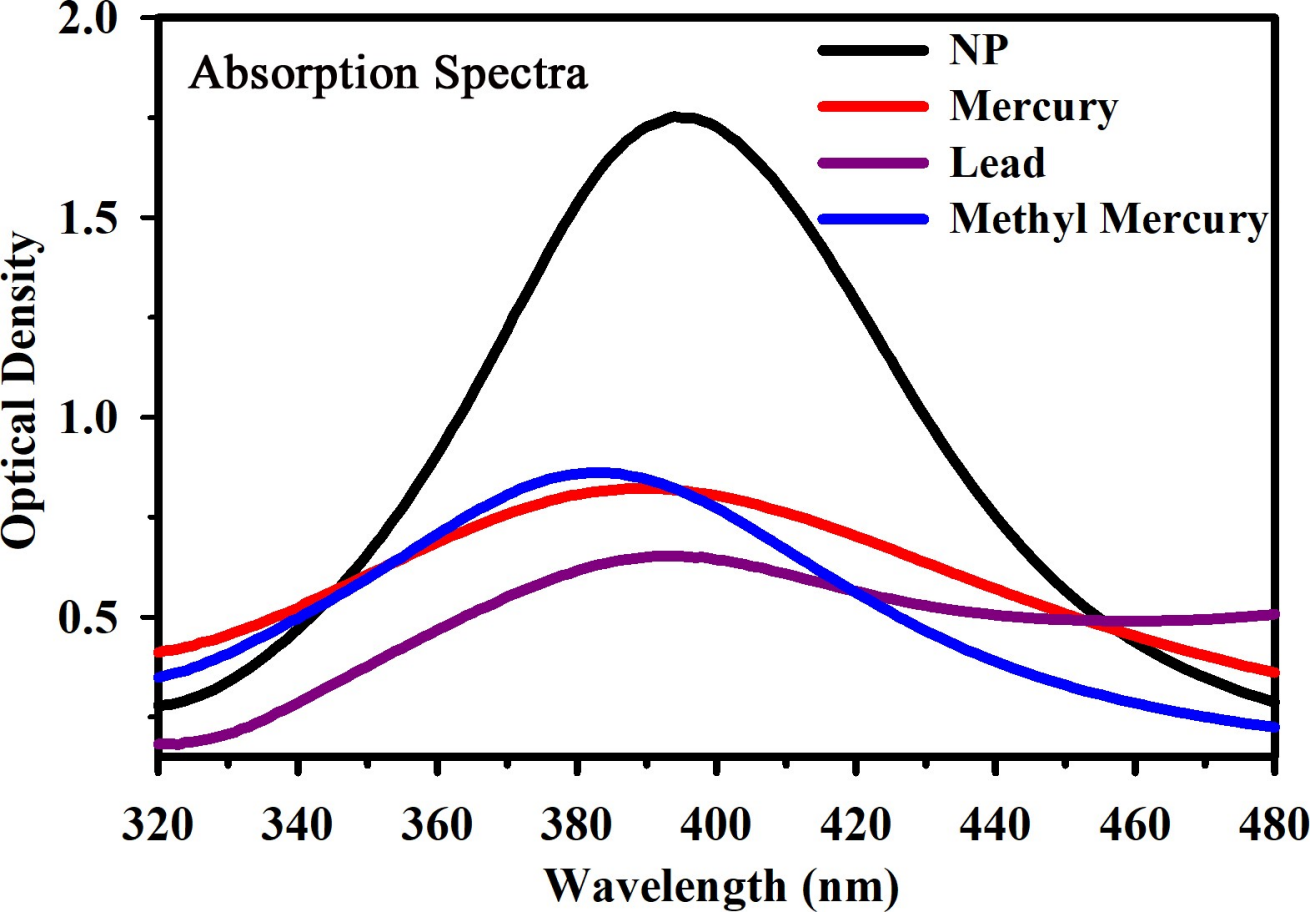
UV-VIS absorption spectra of the Ag NPs before and after their interaction with mercury, lead, and methylmercury in aqueous solution.

**Fig 6:**
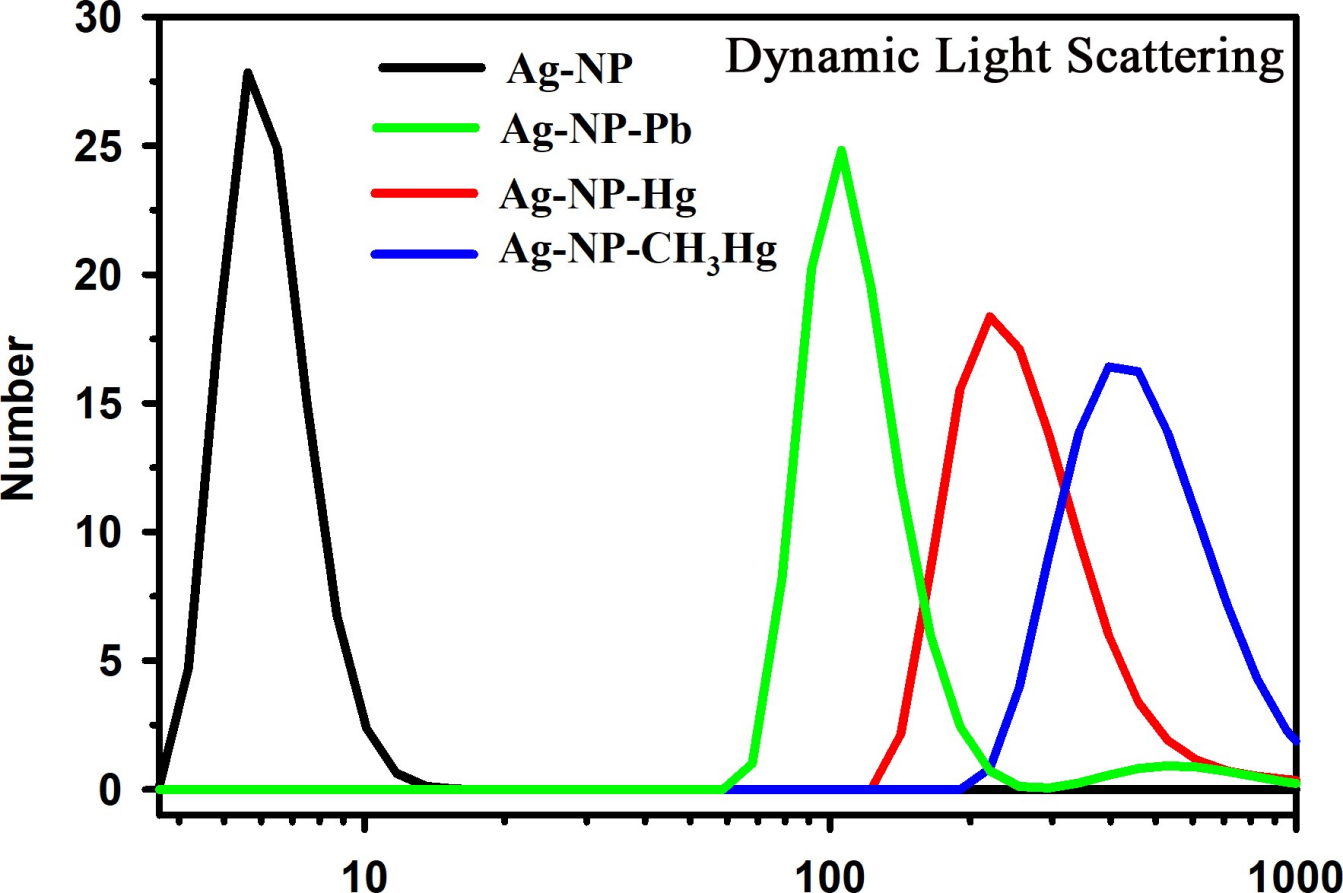
DLS of the Ag NPs before and after interaction with various model pollutants including mercury, lead, and methylmercury in aqueous solution.

Optical absorption spectrometry and high-resolution microscopy were employed to characterise the prepared Ag NP. Fig 4 displays a TEM image of the citrate-capped Ag NPs (a) before and (b) after interaction with the Hg ions in aqueous solution. The change in diameter is due to Hg-induced agglomeration. The Ag NPs were found to be sphere-shaped and distributed uniformly throughout the solution. From the TEM image, the mean radius of the particles was calculated to be approximately 5 nm. Fig 4B illustrates the microscopic data of the NPs with single directional continuous lattice fringes. The inter-planar separation of the Ag NPs was estimated to be approximately 0.23 nm. Fig 5 depicts the absorption spectra of the Ag NP before and after interaction with various model pollutants, including mercury, lead, and methylmercury in aqueous solution. A clear absorbance band peak can be observed from the figure at approximately 395 nm. Dynamic light scattering (DLS) was performed to assess the expected growth of the NPs diameter owing to the addition of mercury, lead, and methylmercury. Fig 6 presents the DLS data of the Ag NPs before and after their interaction with the various model pollutants in aqueous solution. It is observed that the change upon interaction with Hg^2+^ ions is consistent with the HRTEM results. The enhanced particle diameter should trigger an enhanced optical scattering mechanism, which indicates the concentration of the pollutants in the aqueous solution.

### Sensing mechanism and toxic metal ions detection

**Fig 7:**
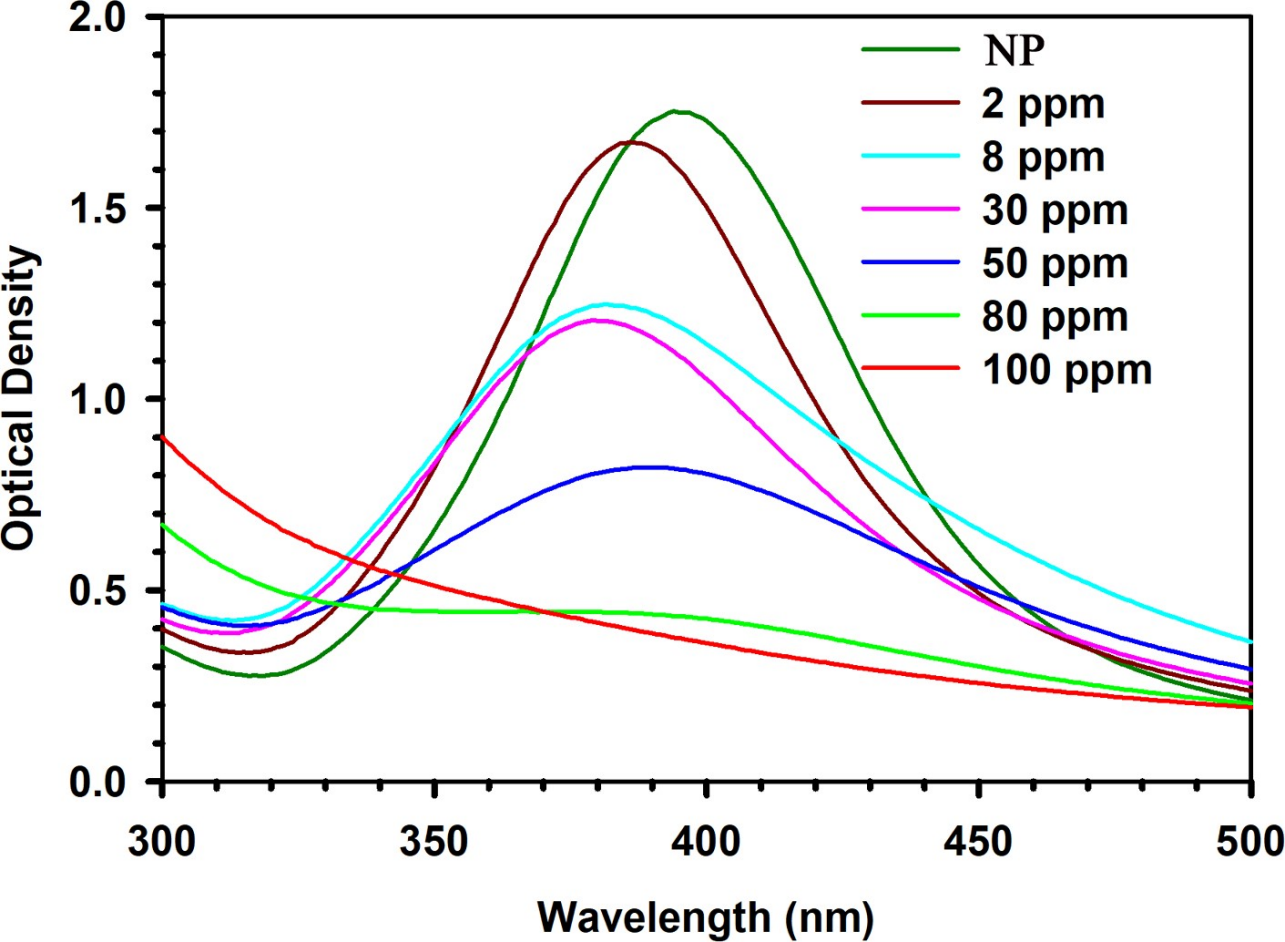
UV-VIS absorption spectra of Ag NPs before and after interaction with various concentrations of mercury ions in aqueous solution.

**Fig 8:**
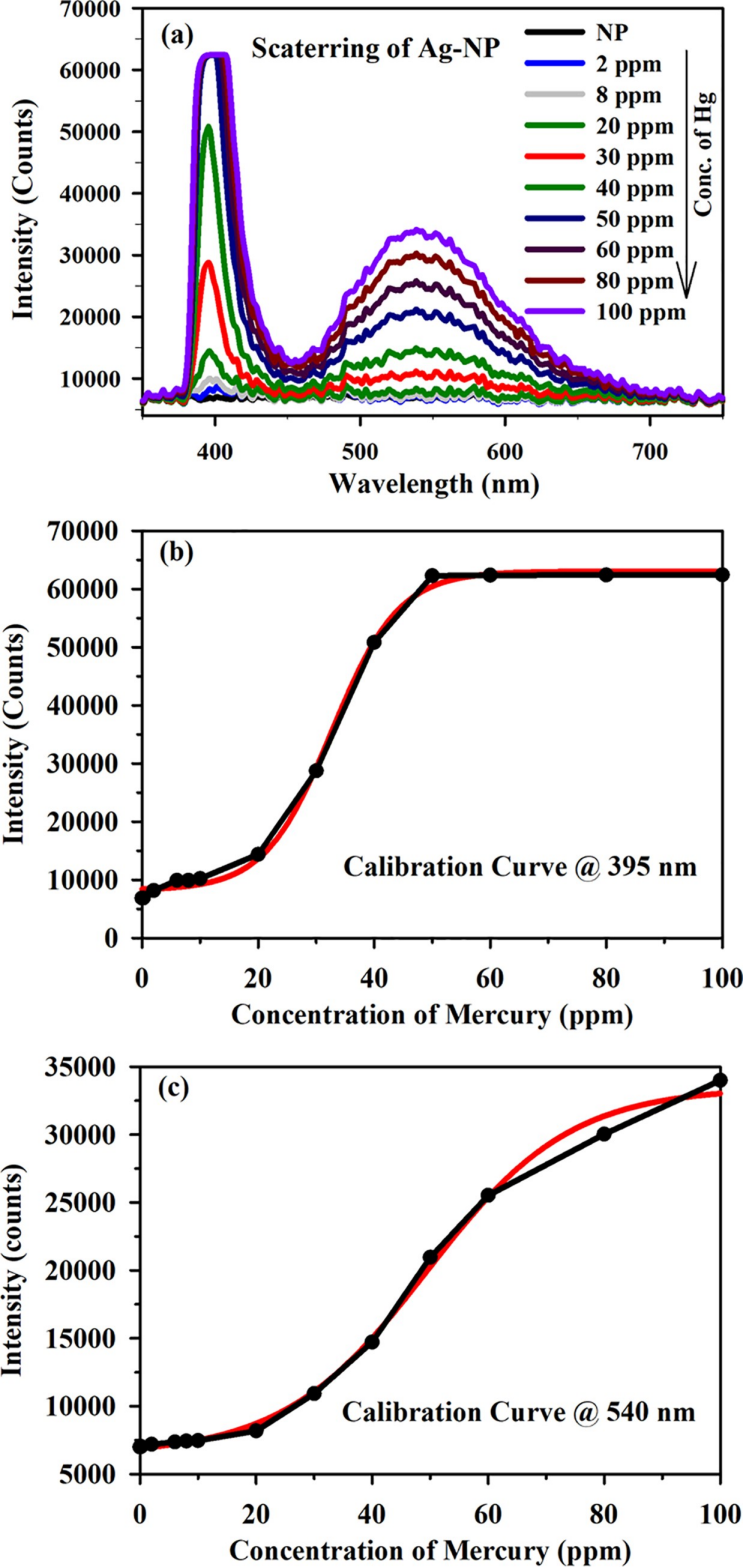
Spectra acquired from the developed device with various concentration of model pollutant (Hg^2+^ ions in water). (a) Post sample emission spectra with peaks at 395 nm (365 nm LED with 50 nm spectral width after 400 nm HP filter) and 520 nm (filter emission). Intensity plot corresponding to various concentrations of Hg^2+^ at (b) 395 nm and (c) 520 nm.

The interaction of the Ag NPs with mercury was investigated by studying the SPR spectra before and after the introduction of the Hg^2+^ aqueous solution. A difference in the ion sensitivity and experimental window is observed for different detection wavelengths. As illustrated in the figure, a minor but consistent reduction in the absorbance was observed within a few seconds of the introduction of the lowest possible mercury concentration of 2 ppm. However, a significant decrease in the spectroscopic peak was observed for higher concentrations of Hg^2+^ ions such as 8 ppm, 30 ppm, 50 ppm, 80 ppm and 100 ppm (Fig 7). (A blue shift in the SPR band was also observed, especially at higher concentrations of Hg^2+^. Fig 7 demonstrates that for few selective concentrations (in the range of 2−100 ppm) of Hg^2+^ ions, the developed sensor exhibits a non-linear nature, which can be described as follows:

$$Y=\frac{X}{\left(10.63+1.4*X-2.66\sqrt{X}\right)},$$ (1)

where Y represents the difference in OD *(I*_*0*_*—I)*, *X* is the Hg^2+^ ion concentration (in ppm), and *I*_*0*_ and *I* are the light intensities of the NPs at 405 nm. The surface amalgamation or improper amalgamation, resulting in smaller NPs, might be attributed to the blue shift of the SPR band. Nevertheless, a complete amalgamation may occur when inducing agglomeration to develop bigger Ag-Hg NPs [36]. In this study, we have developed an optical technique combining Rayleigh scattering and post-sample fluorescence detection from colloidal Ag NPs having SPR band at 420 nm. The spectra acquired from the developed device with various concentration of model pollutant (Hg^2+^ ions in water) were observed. Fig 8(A) illustrates the post sample emission spectra with peaks at 395 nm (365 nm LED with 50 nm spectral width after 400 nm HP filter) and 520 nm (filter emission). Fig 8(B) and 8(C) depict the intensity plots corresponding to various concentrations of Hg at 395 and 520 nm, respectively. The sensitivity of the developed sensor is exceptionally high because the Rayleigh scattering depends on the sixth power of the particle size. The Ag NPs were seen to increase in size with increasing concentration of the metal ions, in the presence of toxic metal ions. Therefore, the sensitivity of our device is very high. The efficacy of the technique is tested for the detection of several toxic ions including mercury, lead, and methylmercury in aqueous media. Fig 8(A) reveals that the light scattering at 395 nm from the Hg-included/inflated Ag NP increased (scattering ‘turn-on’) with a concentration of mercury ions as low as 2 ppm. However, with higher concentrations of mercury ions, saturation occurred due to the large increase in the particle size, which indicates that the developed technique is highly efficient and sensitive (Fig 8(A)). The saturation effect was also predicted by the calibration equation (Eq 2) above a 50 ppm concentration, as shown in Fig 8B.

Y=A2−A11+eX−X0dx (2)

**Fig 9:**
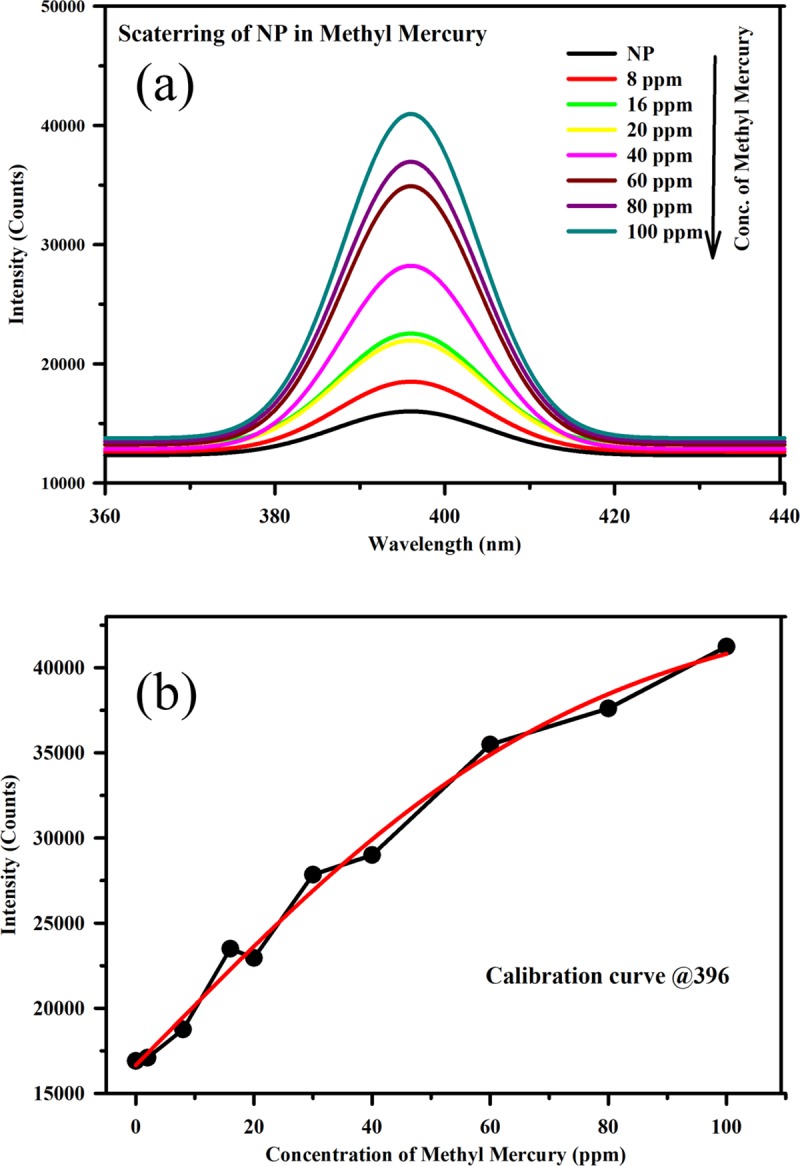
(a) Scattering spectra from the Ag NP with various concentrations of methylmercury. (b) Intensity plot corresponding to various concentrations of methylmercury at 395 nm.

**Fig 10:**
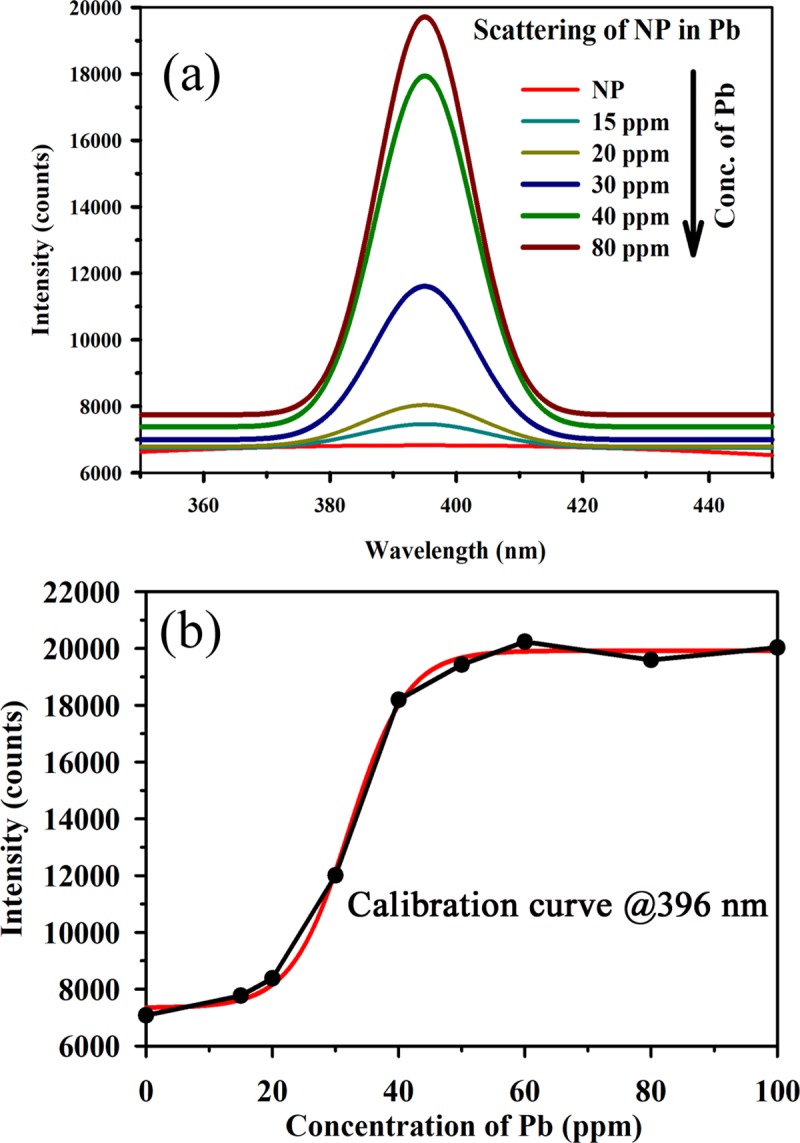
(a) Scattering spectra from the Ag NP with various concentrations of lead. (b) Intensity plot corresponding to various concentrations of lead at 395 nm.

Where A_2_ and A_1_ denotes the final and initial values respectively. X_0_ denotes the centre and dx denotes the time constant. The calibration equation reveals that the developed sensor can detect mercury ions in water medium at concentrations as low as 2 ppm. As mentioned above, the developed sensor cannot determine higher concentrations of pollutants efficiently. For the measurement of higher concentrations of pollutants in water medium, we use the fluorescence of the HP filter (cut-off at 400 nm) at 520 nm. The (High Pass) HP filter fluorescence is effective in determining concentrations of up to several hundreds of ppm. We have also monitored the concentrations of lead and methylmercury and validated the efficacy of the novel strategy for these pollutants. Figs 9(A) and 10(A) illustrate the scattering spectra of the Ag NP with methylmercury and lead, respectively, at various concentrations. Figs 9(B) and 10(B) represent the intensity plots corresponding to various concentrations of methylmercury and lead at 395 nm. It can be observed from the figures that the scattering intensities increase with increasing concentrations of lead and methylmercury. These results demonstrated that the developed technique possesses excellent potential for the detection of lead and methylmercury in aqueous media. The selectivity of the developed sensor in presence of heavy metal ions was found to be effective as discussed in one of our previous publications [34]. However, the shelf life of the Ag nanoparticle-based sensors was found to be approximately one month and hence their use is limited to the mentioned time window only.

## Conclusions

In summary, we have demonstrated that the ‘turn-on’ Rayleigh scattering relies on the specific interaction of functionalized Ag NPs (SPR at 420 nm) with several model water pollutants, including mercury, methylmercury, and lead. It can be an efficient alternative strategy for the development of NP-based sensors with enhanced sensitivity in detection with wider detection window. The decreasing SPR band of the sensor’s NPs indicated the interaction of the sensor with the model pollutants, and high-resolution electron microscopy and enhanced Rayleigh scattering of 356 nm excitation light indicated the enlargement of the NPs due to agglomeration. The post sample HP filter offers significant fluorescence upon receiving scattered light at 365 nm, which was shown to be useful for the detection of higher concentrations of pollutant in the test water. We have also using the above strategy to develop a prototype for the detection of model pollutants and demonstrated its efficacy. Furthermore, the so-called Faraday-Tyndall effect was proved to be useful to develop novel NP-based sensors for monitoring model pollutants.

## References

[1] Lan M, Zhang J, Chui Y-S, Wang P, Chen X, Lee C-S, et al (2014) Carbon nanoparticle-based ratiometric fluorescent sensor for detecting mercury ions in aqueous media and living cells. ACS applied materials & interfaces 6: 21270–21278. 25393954 10.1021/am5062568

[2] Costas-Mora I, Romero V, Lavilla I, Bendicho C (2014) In situ building of a nanoprobe based on fluorescent carbon dots for methylmercury detection. Analytical chemistry 86: 4536–4543. 10.1021/ac500517h 24678836

[3] Renzoni A, Zino F, Franchi E (1998) Mercury levels along the food chain and risk for exposed populations. Environmental Research 77: 68–72. 10.1006/enrs.1998.3832 9600797

[4] Boening DW (2000) Ecological effects, transport, and fate of mercury: a general review. Chemosphere 40: 1335–1351. 10.1016/s0045-6535(99)00283-0 10789973

[5] Tchounwou PB, Ayensu WK, Ninashvili N, Sutton D (2003) Environmental exposure to mercury and its toxicopathologic implications for public health. Environmental Toxicology: An International Journal 18: 149–175. 10.1002/tox.10116 12740802

[6] Langford N, Ferner R (1999) Toxicity of mercury. Journal of human hypertension 13: 651 10.1038/sj.jhh.1000896 10516733

[7] Kessler R (2013) The Minamata Convention on Mercury: a first step toward protecting future generations. National Institute of Environmental Health Sciences. 10.1289/ehp.121-A304 PMC3801463 24218675

[8] Díez S, Delgado S, Aguilera I, Astray J, Pérez-Gómez B, Torrent M, et al (2009) Prenatal and early childhood exposure to mercury and methylmercury in Spain, a high-fish-consumer country. Archives of environmental contamination and toxicology 56: 615–622. 10.1007/s00244-008-9213-7 18836676

[9] Morel FM, Kraepiel AM, Amyot M (1998) The chemical cycle and bioaccumulation of mercury. Annual review of ecology and systematics 29: 543–566.

[10] Castoldi AF, Coccini T, Ceccatelli S, Manzo L (2001) Neurotoxicity and molecular effects of methylmercury. Brain research bulletin 55: 197–203. 10.1016/s0361-9230(01)00458-0 11470315

[11] Yorifuji T, Tsuda T, Harada M (2013) Minamata disease: a challenge for democracy and justice Late Lessons from Early Warnings: Science, Precaution, Innovation Copenhagen, Denmark: European Environment Agency.

[12] Tz Guo, Baasner J, Gradl M, Kistner A (1996) Determination of mercury in saliva with a flow-injection system. Analytica chimica acta 320: 171–176.

[13] Wang H-T, Kang B, Chancellor T Jr, Lele T, Tseng Y, Ren F, et al (2007) Fast electrical detection of Hg (II) ions with Al Ga N∕ Ga N high electron mobility transistors. Applied Physics Letters 91: 042114.

[14] Leopold K, Foulkes M, Worsfold P (2010) Methods for the determination and speciation of mercury in natural waters—a review. Analytica chimica acta 663: 127–138. 10.1016/j.aca.2010.01.048 20206001

[15] Bernaus A, Gaona X, Esbrí JM, Higueras P, Falkenberg G, Valiente M (2006) Microprobe techniques for speciation analysis and geochemical characterization of mine environments: the mercury district of Almadén in Spain. Environmental science & technology 40: 4090–4095. 16856721 10.1021/es052392l

[16] Senapati T, Senapati D, Singh AK, Fan Z, Kanchanapally R, Ray PC (2011) Highly selective SERS probe for Hg (II) detection using tryptophan-protected popcorn shaped gold nanoparticles. Chemical Communications 47: 10326–10328. 10.1039/c1cc13157e 21853207

[17] Chen Y, Wu L, Chen Y, Bi N, Zheng X, Qi H, et al (2012) Determination of mercury (II) by surface-enhanced Raman scattering spectroscopy based on thiol-functionalized silver nanoparticles. Microchimica Acta 177: 341–348.

[18] Wu J, Liu W, Ge J, Zhang H, Wang P (2011) New sensing mechanisms for design of fluorescent chemosensors emerging in recent years. Chemical Society Reviews 40: 3483–3495. 10.1039/c0cs00224k 21445455

[19] Lan M, Wu J, Liu W, Zhang W, Ge J, Zhang H, et al (2012) Copolythiophene-derived colorimetric and fluorometric sensor for visually supersensitive determination of lipopolysaccharide. Journal of the American Chemical Society 134: 6685–6694. 10.1021/ja211570a 22452659

[20] Lan M, Liu W, Wang Y, Ge J, Wu J, Zhang H, et al (2013) Copolythiophene-derived colorimetric and fluorometric sensor for lysophosphatidic acid based on multipoint interactions. ACS applied materials & interfaces 5: 2283–2288. 23459452 10.1021/am400319g

[21] Miao R, Mu L, Zhang H, She G, Zhou B, Xu H, et al (2014) Silicon nanowire-based fluorescent nanosensor for complexed Cu2+ and its bioapplications. Nano letters 14: 3124–3129. 10.1021/nl500276x 24837483

[22] Lee MH, Kim HJ, Yoon S, Park N, Kim JS (2008) Metal ion induced FRET OFF− ON in Tren/Dansyl-appended rhodamine. Organic letters 10: 213–216. 10.1021/ol702558p 18078343

[23] Lee MH, Van Giap T, Kim SH, Lee YH, Kang C, Kim JS (2010) A novel strategy to selectively detect Fe (III) in aqueous media driven by hydrolysis of a rhodamine 6G Schiff base. Chemical Communications 46: 1407–1409. 10.1039/b921526c 20162130

[24] Costa-Fernández JM, Pereiro R, Sanz-Medel A (2006) The use of luminescent quantum dots for optical sensing. TrAC Trends in Analytical Chemistry 25: 207–218.

[25] Frasco M, Chaniotakis N (2009) Semiconductor quantum dots in chemical sensors and biosensors. Sensors 9: 7266–7286. 10.3390/s90907266 22423206 PMC3290488

[26] Freeman R, Willner I (2012) Optical molecular sensing with semiconductor quantum dots (QDs). Chemical Society Reviews 41: 4067–4085. 10.1039/c2cs15357b 22481608

[27] Yu S-Y, Chen Y-J, Liaw J-W. Faraday-Tyndall effect of gold colloids; 2015 IEEE. pp. 1–3.

[28] Liaw J-W, Tsai S-W, Lin H-H, Yen T-C, Chen B-R (2012) Wavelength-dependent Faraday–Tyndall effect on laser-induced microbubble in gold colloid. Journal of Quantitative Spectroscopy and Radiative Transfer 113: 2234–2242.

[29] Tripathi KM, Tran TS, Kim YJ, Kim T (2017) Green fluorescent onion-like carbon nanoparticles from flaxseed oil for visible light induced photocatalytic applications and label-free detection of Al (III) ions. ACS Sustainable Chemistry & Engineering 5: 3982–3992.

[30] Tripathi KM, Singh A, Myung Y, Kim T, Sonkar SK (2018) Sustainable nanocarbons as potential sensor for safe water. Nanotechnology for sustainable water resources 1: 141–176.

[31] Andjelkovic I, Tran DN, Kabiri S, Azari S, Markovic M, Losic D (2015) Graphene aerogels decorated with α-FeOOH nanoparticles for efficient adsorption of arsenic from contaminated waters. ACS applied materials & interfaces 7: 9758–9766. 25871444 10.1021/acsami.5b01624

[32] Zhang G, Liu M (2000) Effect of particle size and dopant on properties of SnO2-based gas sensors. Sensors and Actuators B: Chemical 69: 144–152.

[33] Velázquez-González JS, Monzón-Hernández D, Moreno-Hernández D, Martínez-Piñón F, Hernández-Romano I (2017) Simultaneous measurement of refractive index and temperature using a SPR-based fiber optic sensor. Sensors and Actuators B: Chemical 242: 912–920.

[34] Sarkar PK, Polley N, Chakrabarti S, Lemmens P, Pal SK (2016) Nanosurface energy transfer based highly selective and ultrasensitive “turn on” fluorescence mercury sensor. ACS Sensors 1: 789–797.

[35] Flores C, Diaz C, Rubert A, Benítez G, Moreno M, de Mele MFL, et al (2010) Spontaneous adsorption of silver nanoparticles on Ti/TiO2 surfaces. Antibacterial effect on Pseudomonas aeruginosa. Journal of Colloid and Interface Science 350: 402–408. 10.1016/j.jcis.2010.06.052 20656295

[36] Sarkar PK, Halder A, Polley N, Pal SK (2017) Development of highly selective and efficient prototype sensor for potential application in environmental mercury pollution monitoring. Water, Air, & Soil Pollution 228: 314.

